# Expression of concern: Picolylamine–Ni(ii) complex attached on 1,3,5-triazine-immobilized silica-coated Fe_3_O_4_ core/shell magnetic nanoparticles as an environmentally friendly and recyclable catalyst for the one-pot synthesis of substituted pyridine derivatives

**DOI:** 10.1039/d5ra90022k

**Published:** 2025-03-07

**Authors:** Sobhan Rezayati, Fatemeh Kalantari, Ali Ramazani

**Affiliations:** a Department of Chemistry, Faculty of Science, University of Zanjan Zanjan 45371-38791 Iran aliramazani@gmail.com aliramazani@znu.ac.ir; b Department of Biotechnology, Research Institute of Modern Biological Techniques (RIMBT), University of Zanjan Zanjan 45371-38791 Iran

## Abstract

Expression of concern for ʻPicolylamine–Ni(ii) complex attached on 1,3,5-triazine-immobilized silica-coated Fe_3_O_4_ core/shell magnetic nanoparticles as an environmentally friendly and recyclable catalyst for the one-pot synthesis of substituted pyridine derivativesʼ by Sobhan Rezayati *et al.*, *RSC Adv.*, 2023, **13**, 12869–12888, https://doi.org/10.1039/D3RA01826A.

The Royal Society of Chemistry is publishing this expression of concern in order to alert readers that concerns have been raised regarding the integrity of the XRD data in Fig. 5.

The Royal Society of Chemistry has asked the affiliated institution to investigate this matter and establish whether the data provided by the authors provide an accurate representation of the experiments that were conducted and to confirm the integrity and reliability of the data provided.

An expression of concern will continue to be associated with the article until we receive conclusive evidence regarding the reliability of the reported data.

Laura Fisher

28th February 2025

Executive Editor, *RSC Advances*

